# Retraction Note: Combined effect of genetic background and gender in a mouse model of bleomycin-induced skin fibrosis

**DOI:** 10.1186/s13075-023-03211-7

**Published:** 2023-11-13

**Authors:** Nadira Ruzehaji, Jerome Avouac, Muriel Elhai, Maxime Frechet, Camelia Frantz, Barbara Ruiz, Joerg H. Distler, Yannick Allanore

**Affiliations:** 1 INSERM U1016/UMR 8104, Cochin Institute, Paris, France; 2 Institut Cochin, INSERM U1016, Bâtiment Gustave Roussy, 27 Rue du Faubourg Saint Jacques, 75014 Paris, France; 3 Rheumatology A Department, Paris Descartes University, Paris, France; 4 Department of Internal Medicine and Institute for Clinical Immunology, University of Erlangen-Nuremberg, Erlangen, Germany

**Retraction Note: Arthritis Res Ther 17, 145 (2015)**

**https://doi.org/10.1186/s13075-015-0659-5**

The Editors-in-Chief has retracted this article because after the publication of the article concerns were raised about image overlap. Further investigation by the Publisher found the following concerns to be valid.

- Partial image overlap between Fig. 1A (upper-left panel, NaCl- BALB/c male mouse) and Fig. 2A (upper-left panel, NaCl—BALB/c female mouse)
- Partial overlap between Fig. 1A (upper-right panel, NaCl- DBA/2 male mouse) and Fig. 2A (upper-right panel, NaCl-DBA/2 female mouse).
- Partial image overlap between Fig. 2A (upper-right panel, NaCl-DBA/2) with Fig. 6A (left panel, NaCl)
- Partial image overlap in Fig. 2A (lower-middle panel, Bleomycin- C57BL/6) with Fig. 5A (upper-right panel, NaCl + Control mAb) of [1].
- Partial image overlap in Fig. 3D (lower-middle panel, Bleomycin C57BL6/2) with Fig. 6B (lower-right panel, KLH BLM) of [2].

The authors provided the raw data for validation. However, further checks by the Publisher identified some differences between the raw data and the figures, as well as overlap in raw images. The authors were unable to provide ethical approval for experiments involving Balb/c mice strain. The Editors-in-Chief therefore no longer has confidence in the presented data.

Nadine Ruzehaji, Muriel Elhai, Jerome Avouac, Camelia Frantz, Joerg H. Distler and Yannick Allanore agree to this retraction. Maxime Frechet has not responded to any correspondence from the editor/publisher about this retraction. The editor was not able to obtain a current email address for Barbara Ruiz.
